# Validation of a light-scattering PM_2.5_ sensor monitor based on the long-term gravimetric measurements in field tests

**DOI:** 10.1371/journal.pone.0185700

**Published:** 2017-11-09

**Authors:** Jingjin Shi, Fei’er Chen, Yunfei Cai, Shichen Fan, Jing Cai, Renjie Chen, Haidong Kan, Yihan Lu, Zhuohui Zhao

**Affiliations:** 1 Department of Environmental Health, School of Public Health, Fudan University, Shanghai 200032, China; 2 International Peace Maternity and Child Health Hospital of China Welfare Institute, Shanghai Jiao Tong University School of Medicine, Shanghai, China; 3 Shanghai Environmental Monitoring Center, Shanghai, China; 4 Department of Environmental Health, Rollins School of Public Health, Emory University, GA, United States of America; 5 Key Laboratory of Public Health Safety of the Ministry of Education, Key Lab of Health Technology Assessment, National Health and Family Planning Commission of the People's Republic of China, Shanghai Key Laboratory of Meteorology and Health, Fudan University, Shanghai, China; 6 Shanghai Key Laboratory of Particle Pollution and Prevention (LAP^3^), Fudan University, Shanghai, China; 7 Department of Epidemiology, School of Public Health, Fudan University, Shanghai, China; Telethon Institute for Child Health Research, AUSTRALIA

## Abstract

**Background:**

Portable direct-reading instruments by light-scattering method are increasingly used in airborne fine particulate matter (PM_2.5_) monitoring. However, there are limited calibration studies on such instruments by applying the gravimetric method as reference method in field tests.

**Methods:**

An 8-month sampling was performed and 96 pairs of PM_2.5_ data by both the gravimetric method and the simultaneous light-scattering real-time monitoring (QT-50) were obtained from July, 2015 to February, 2016 in Shanghai. Temperature and relative humidity (RH) were recorded. Mann-Whitney U nonparametric test and Spearman correlation were used to investigate the differences between the two measurements. Multiple linear regression (MLR) model was applied to set up the calibration model for the light-scattering device.

**Results:**

The average PM_2.5_ concentration (median) was 48.1μg/m^3^ (min-max 10.4–95.8μg/m^3^) by the gravimetric method and 58.1μg/m^3^ (19.2–315.9μg/m^3^) by the light-scattering method, respectively. By time trend analyses, they were significantly correlated with each other (Spearman correlation coefficient 0.889, P<0.01). By MLR, the calibration model for the light-scattering instrument was Y(calibrated) = 57.45 + 0.47 × X(the QT – 50 measurements) – 0.53 × RH – 0.41 × Temp with both RH and temperature adjusted. The 10-fold cross-validation R^2^ and the root mean squared error of the calibration model were 0.79 and 11.43 μg/m^3^, respectively.

**Conclusion:**

Light-scattering measurements of PM_2.5_ by QT-50 instrument overestimated the concentration levels and were affected by temperature and RH. The calibration model for QT-50 instrument was firstly set up against the gravimetric method with temperature and RH adjusted.

## Introduction

Airborne particulate matter with the aerodynamic diameter equal to or less than 2.5μm(PM_2.5_) has been linked to respiratory or cardiovascular diseases and all-cause mortality in epidemiological studies worldwide including developing countries [1–4]. Exposure assessment is crucial for the accurate estimate on the PM_2.5_ health effects. Many previous epidemiological studies relied on the ground fixed-site monitoring stations[5, 6] and were unavoidably with potential exposure misclassification. The monitoring of PM_2.5_ in microenvironment or by personal monitoring is a good supplement to the ambient fixed-site monitoring. However, by a lack of calibration on these portable PM_2.5_ instruments, their accuracy and reliability are often unknown which limit their application in scientific research.

The portable and direct-reading PM_2.5_ instruments are generally based on the light-scattering and particle absorbance theories[7]. The particle number concentration is counted and then transferred to mass concentration as the output. The measurements of particles by light-scattering method, however, are often affected by water vapor or droplets in the air which can be assessed by measuring the high relative humidity (RH). Previous results showed that light-scattering technology tends to overestimate particulate levels when compared with the gravimetric method, especially under higher RH [8–13]. Other factors such as the particle size distribution, particle morphology and chemical constituents also influence the measurements of PM_2.5_, of which the influence magnitude may vary by different pollution sources. So it is necessary to set up the calibration model for the light-scattering instrument based on the local long-time sampling with temperature and RH adjusted. By comparing with the filter-based gravimetric measurements, regarded as the reference standard method, the calibration model was constructed in this study based on an 8-month parallel field test between the light-scattering method and the gravimetric method.

The objectives of this study were to set up the calibration model by investigating the relationship between the measurements by the light-scattering PM_2.5_ monitor (QT-50, Hivron, Beijing, China) and by the parallel gravimetric measurements, and further establish the method of field calibration for low-cost optical sensors. We are going to investigate the factors influencing the differences between the two sets of measurement data and eventually construct a validation model for the PM_2.5_ sensor instrument with temperature and RH adjusted, based on long-time field tests. Because the temperature and RH are largely different between indoor and outdoor environment, both indoor and outdoor samplings and comparisons were conducted at the same time to enable the validation model be applicable in a variety of environment.

## Materials and methods

### PM_2.5_ sensor instrument

**Fig 1:**
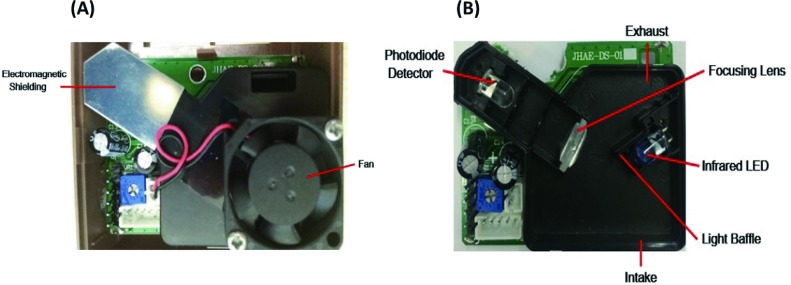
Schematic designs of QT-50 (Hivron, Beijing, China) (a) with electromagnetic shielding and fan (b) without electromagnetic shielding and fan.

The light-scattering PM_2.5_ sensor instruments (QT-50, Hivron, Beijing, China) were pocket-size (weight<300g, volume<510cm^3^). It consisted of a PM sensor (DS-01D-V1), a microprocessor, a real-time clock, a data logger, a temperature and relative humidity sensor, a network module, and a small light emitting diode (LED) display screen. In the PM_2.5_ monitoring sensor(DS-01D-V1), an infrared light emitting diodes at 650 nm was used as the light source and a photodiode detector was used as the detector for scattered lights at a scattering angle of 90°(Patent number: CN201430196873.5). The schematics of QT50 was presented in Fig 1 as follows. QT-50 has a fan installed for a better airflow. This is different with some other sensors, such as Shinyei and Samyoung[14, 15]. The Shinyei and Samyoung PM sensors were on the principle by electrically heating a resistor near the sensor inlet to sample particles through sensing volumes. In this study, QT-50 instruments were connected to 220 V wall outlet power during the long-term monitoring.

### RH-calibrated PM_2.5_ sensor

The MicroPEM (RTI International, Research Triangle Park, NC, USA) is used as a RH-calibrated light-scattering instrument in this study. MicroPEM is a wearable PM personal exposure monitor based on laser light-scattering method, which is able to monitor the PM mass concentration both in a real-time mode and in collecting PM_2.5_ samples on filters. The readout of MicroPEM is based on its build-in calibration curve, the principles and validation of this instrument have been given in detail elsewhere [16–18]. MicroPEM was fitted with a two-stage mini-impactor set with initial and final cut points at 4.0μm and 2.5μm, respectively. Before each sampling, MicroPEM was zeroed with an in-line HEPA filter, and pre-calibrated at 0.50 LPM by a TSI model 4100 mass flowmeter (TSI, Inc., Shoreview, MN, USA) by using Docking Station software (RTI International, Research Triangle Park, NC, USA). MicroPEM overcomes the disturbance of water droplets or water vapor by applying the internal RH correction coefficient.

### Gravimetric filter sampling

SKC pumps (Aircheck Sampler, Model 224-PCXR8, SKC Inc., PA, USA) were used to collect the PM_2.5_ samples using a 2.5μm impactor (SKC Inc., PA, USA). A flow rate calibration chamber (SKC Gulf Coast Inc., Houston, TX, USA) was used before and after the sampling to facilitate the measurements of the flow rate (Liter per minute, LPM). For each sampling, a 37mm Quartz filter (Pall Corp., Ann Arbor, MI, USA) was pre-heated at 900°C for 3h to remove carbon contamination. All Quartz filters were pre- and post-weighed in a temperature and RH constant room using a Mettler-Toledo AG285 electronic microbalance with ±0.01mg sensitivity. Filters were repeatedly weighed until the differences between replicate weights were <20 mg and <10 mg for samples and blanks, respectively. The number of field blank samples (which were placed into the filter holders in the field but did not sample any air) accounted for 15% of all gravimetric samples. The integrated gravimetric PM_2.5_ mass concentration was calculated by dividing the net mass changes(μg) by the total air volume sampled(m^3^). To minimize the evaporation of volatile components, sampled filters were stored at 4°C until analysis.

$$\rho =\frac{w_{2}-w_{1}}{V_{n}}x1000$$ (1)

ρ:Average particulate matter mass concentration, μg/m^3^;
w1:Mass of the membrane before sampling, μg;
w2:Mass of the membrane after sampling, μg;
Vn:Sampling volume transformed in standard state(273.15K,101.325KPa), L.

### Experimental setting

The PM_2.5_ concentrations were monitored by 3 types of instruments for 8 continuous months (from July, 2015 to February, 2016) both indoor and outdoor in 3 representative locations. Totally 96 measurements were completed, twice a month in each location both indoor and outdoor simultaneously (2 x 8 x 2 x 3 = 96).

The 3 types of instruments were 1) PM_2.5_ sensor instrument (QT-50), 2) PM_2.5_ sensor reference instrument (MicroPEM) and 3) the gravimetric method (by SKC pumps) as described above. To be representative of the ordinary indoor environment, the 3 locations were selected in the urban area of Shanghai: 1) a university office in the Jiangwan Campus of Fudan University, 2) a 4-person dormitory room in the Jiangwan Campus of Fudan University and 3) a residential building (on the 8^th^ floor), 2 km far from Jiangwan Campus of Fudan University. All three instruments were put in the same locations at the same height to the floor. Temperature and RH during the whole sampling were measured and recorded by the HOBO data logger (U12-012, Onset Computer Corporation, Pocasset, MA, USA).

For the two filer samples in each location in each month, one sample was performed in 4 continuous weekdays (the inlet flow rate was 2L/min) and the other was in the weekend for 2 continuous days (the inlet flow rate was 4L/min matched with the impactor). There were eventually 54 filter samples of PM_2.5_ obtained successfully excluding the missing data due to instrumental failure or filter membrane damage. For the PM_2.5_ sensor instruments (QT-50 and MicroPEM), data were continuously monitored and recorded every 10min. According to the beginning and ending time of the filter sampling, the corresponding real-time measurement data by QT-50 or MicroPEM were calculated into the 2-day or 4-day average values in order to be matched and comparable with the integrated average value by the gravimetric method. The data in 3 locations were polled together to set up the calibration model of QT-50 instrument.

### Statistical analyses

The original real-time monitoring data (every 10 min) by QT-50 and MicroPEM were firstly calculated into the 24-hour average level. The 24-hour average data were then paired by time between the two sensor instruments. For the comparisons between the sensor monitoring data and the gravimetric data, the 24-hour sensor monitoring data were further calculated into the integrated average level corresponding to each specific sampling time period of the gravimetric sampling as mentioned above. Eventually 75 pairs of data were obtained between the QT-50 instrument and the gravimetric method and 54 groups of complete data were obtained with all 3 types of instruments.

Mann-Whitney U nonparametric test was used to compare the differences between 3 groups of data since PM_2.5_ concentrations were abnormally distributed. Spearman correlation analyses were performed between any two measurements of QT-50, MicroPEM and gravimetric measurement data. The calibration model for QT-50 was set up by multiple linear regression with the gravimetric measurements (the reference method) as the dependent variable and QT-50 data as the independent variables, adjusted for temperature and RH. The similar model was set up for MicroPEM as for a comparison. The 10-fold cross-validation (10-fold CV) was performed to validate the multiple linear regression model for QT-50. The overall fit R^2^ and root mean squared error (RMSE) between the predicted and measured concentrations of CV were calculated to evaluate the model performance [19]. All analyses were conducted with R software (Version 2.15.3, R Development Core Team) and SPSS (Version 22.0, IBM).

## Results

In the 8-month sampling, the indoor median level of PM_2.5_ was 47.8 μg/m^3^ (19.2–135.1μg/m^3^) by the real-time QT-50 sensor instrument, 36.7 μg/m^3^ (11.7–142.3μg/m^3^) by the real-time MicroPEM instrument and 39.7 μg/m^3^(10.4–95.8μg/m^3^) by the gravimetric method (Table 1). The indoor levels measured by QT-50 were higher than the ones by MicroPEM and the gravimetric method. For outdoor air PM_2.5_, the ranking of the concentrations by the 3 instruments were similar with the indoor ones. By polling the indoor and outdoor measurement data together, QT-50 measurements were significantly higher than MicroPEM and the gravimetric method. In the whole sampling, the median indoor temperature and RH was 20.5°C and 61.6% and the outdoor ones were 14.4°C and 54.3%.

**Table 1:** PM_2.5_ mass concentration(μg/m^3^)(median, range) measured by the real-time sensor instruments(QT-50 and MicroPEM) and the gravimetric method as well as the temperature and RH throughout the sampling period (based on the matched 54 groups of data by 3 instruments).

	Total (n = 54)	Indoor (n = 36)	Outdoor (n = 18)
**QT-50 light-scattering sensor**	58.1* ^a^ (19.2–315.9)	47.8^NS^ ^a^ (19.2–135.1)	82.4* ^a^ (34.0–315.9)
**MicroPEM light-scattering sensor**	40.3* ^b^ (11.7–214.1)	36.7 ^NS^ ^b^ (11.7–142.3)	64.2 ^NS^ ^b^ (30.0–214.1)
**Gravimetric method by SKC pump**	48.1 (10.4–159.8)	39.7 (10.4–95.8)	55.9 (16.8–159.8)
**Temp(°C)**	19.3 (7.0–32.5)	20.5 (6.97–32.5)	14.4 (7.18–27.8)
**RH(%)**	63.0 (44.1–90.2)	61.6 (44.1–81.1)	54.3 (66.9–90.2)

Comparison between two light-scattering sensors and gravimetric measurement, respectively

^a^ refers to the comparisons between QT-50 and the gravimetric method.

^b^ refers to the comparisons between MicroPEM and gravimetric method.

* *P*<0.05

NS, non-significance

### Correlations between QT-50, MicroPEM and the gravimetric method

**Fig 2:**
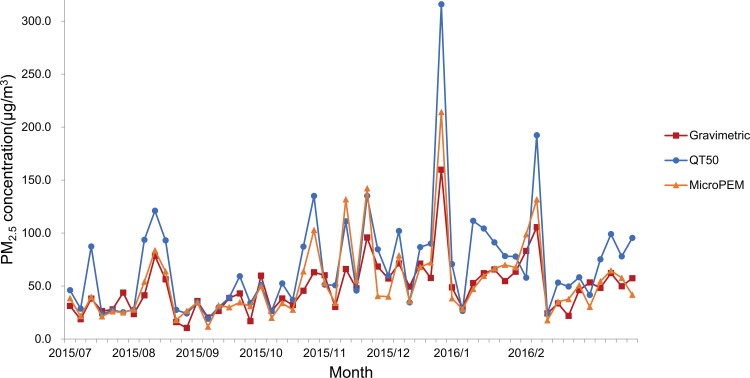
Time trend graph for PM_2.5_ levels measured by QT-50, MicroPEM and the gravimetric method. To evaluate and validate QT50 for PM2.5 in field measurements, parallel sampling was performed by QT50, an established light-scattering instrument (MicroPEM) and by gravimetric method. QT50 and MicroPEM recorded PM2.5 data for every 10 min continuously and the filters were sampled for 2 days in the weekend or 4 days in weekdays. According to the beginning and ending time of the filter sampling, the real-time measurement data by QT-50 or MicroPEM were calculated into the 2-day or 4-day average values to be matched and comparable with the integrated average value by the gravimetric method. Eventually, 54 pairs of matched values were obtained and presented in Fig 2. The date points refer to each paired average values. Y-axis refers to PM2.5 average levels and X-axis refers to sampling date.

There were high correlations between the measurements by QT-50, MicroPEM and the gravimetric method. By comparing the 54 groups of matched data by 3 instruments, the spearman correlation coefficients were 0.891 (P = 0.0001) between QT-50 and MicroPEM (the hourly average data, n = 6549), 0.817 (P = 0.0001) between QT-50 and the gravimetric data (n = 54) and 0.875 (P = 0.0001) between MicroPEM and the gravimetric method (n = 54) (Fig 2).

**Fig 3:**
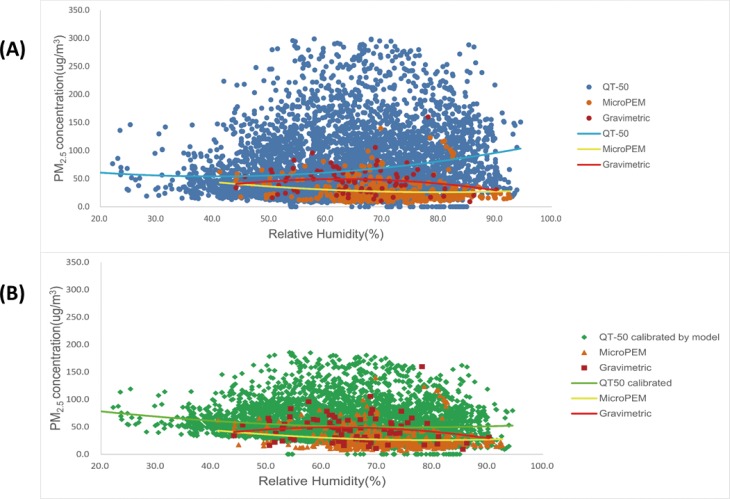
**The distribution of PM**_**2.5**_ **concentration by QT-50, MicroPEM and gravimetric method at different RH before QT-50 calibration (A) and after calibration (B). **In both Fig 3(A) and Fig 3(B), each point refers to a PM_2.5_ concentration data and the lines refer to the PM_2.5_ trend by RH. The points by QT-50 and MicroPEM were the original 10-min data of PM_2.5_. The points indicated as “Gravimetric” were the integrated average in each sample by SKC pumps. Y axis refers to the PM_2.5_ concentration level (μg/m^3^) and X axis refers to RH.

**Fig 4:**
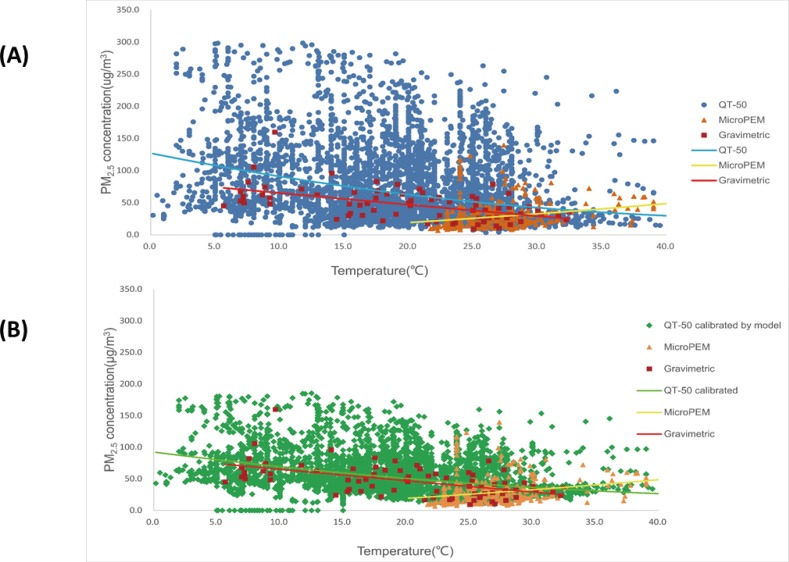
**The distribution of PM**_**2.5**_ **concentrations by QT-50, MicroPEM and gravimetric methods at different temperatures before QT-50 calibration (A) and after calibration (B).** In both Fig 4A and 4B, each point refers to a PM_2.5_ concentration data and the lines refer to the PM_2.5_ trend by temperature. The points by QT-50 and MicroPEM were the original 10-min data of PM_2.5_. The points indicated as “Gravimetric” were the integrated average in each sample by SKC pumps. Y axis refers to the PM_2.5_ concentration level (μg/m^3^) and X axis refers to temperature.

When analyzing the data distribution of PM_2.5_ by temperature and RH, it was found that (Fig 3A), with the increase of RH in the air, there was a significant increase of PM_2.5_ by QT-50. However, no such a trend was observed in the data by MicroPEM and the gravimetric method. On the other hand, with the increase of temperature, no obvious trend was observed for PM_2.5_ (Fig 4A).

### Calibration of the QT-50 instrument

Between the QT-50 instrument and the gravimetric method, a total of 75 pairs of data were obtained (S1 File). In comparison with the gravimetric data, QT-50 measurements were on average 1.51 (SD 0.66, range 0.58–4.46) times of the gravimetric data. In order to find out the appropriate calibration model for QT-50, a stepwise linear regression was performed. Firstly, the basic regression model was set up with the gravimetric data as the Y and QT-50 data as the X. In order to adjust the influence of RH on PM_2.5_, the second regression was performed with RH added in the basic model, and finally, both RH and temperature were added in the regression model (Table 2).

**Table 2:** The stepwise linear calibration models with adjusted R^2^ and 10-fold cross validation (10-fold CV) R^2^ and coefficients.

Model	N	Model R^2^	10-fold CV R^2^	Coefficients(β)			
		(RMSE)	(RMSE)	Intercept	QT-50 (μg/m^3^)	RH (%)	Temp (°C)
**Basic model**	75	0.75 (12.44)	0.73 (12.76)	14.15	0.48	/	/
**Basic model + RH**	75	0.79 (11.17)	0.78 (11.56)	50.65	0.50	-0.58	/
**Basic model + RH +T**	75	0.80 (10.87)	0.79 (11.43)	57.45	0.47	-0.53	-0.41

There were 75 matched pairs of data by the QT-50 and the gravimetric method. A higher R^2^ and a lower RMSE indicated a better fitting model.

The basic regression model was obtained as follows

Y=0.48×X+14.1 (2)

In this model, the R^2^ was 0.75 and the 95%CI of the slope (0.48) was 0.42–0.55. Compared with the theoretically ideal measurements, e.g. Y = X, it demonstrated that the differences (d = 0.52x-14.15) between the gravimetric and the QT-50 data were from both the system bias but also associated with the PM_2.5_ concentration levels.

With RH and temperature added in, the R^2^ and the 10-fold CV R^2^ of the model increased to 0.80 and 0.79, respectively, while the RMSE between the predicted and measured concentrations of CV decreased to 10.87μg/m^3^ and 11.43μg/m^3^, respectively (Table 2). A higher R^2^ and a lower RMSE R^2^ was obtained, which indicated a more fit regression model was achieved. Therefore, the QT-50 calibration model was determined as follows:

Y=57.45+0.47×X−0.53×RH−0.41×Temp (3)

where Y refers to the calibrated PM_2.5_ levels and X was the original PM_2.5_ measured values by the QT-50 monitor.

### QT-50 data comparisons before and after RH calibration

**Fig 5:**
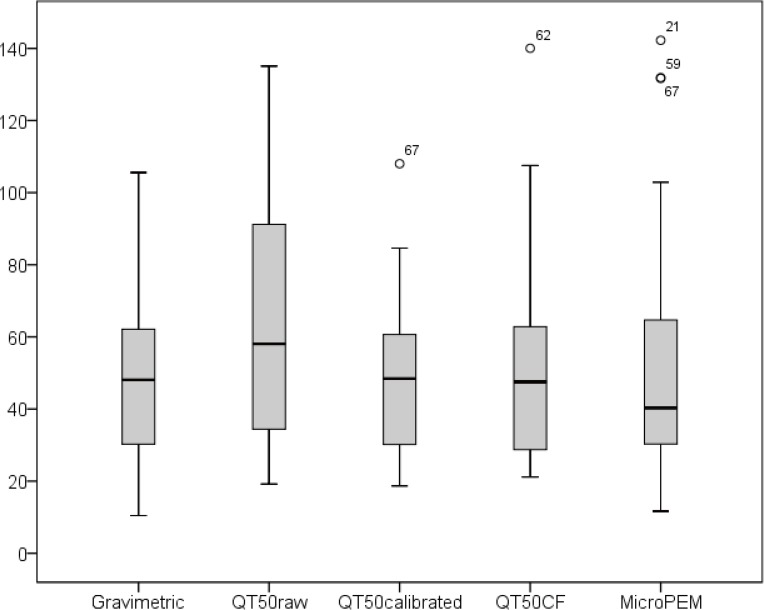
Comparisons of two light-scattering devices (QT-50 and MicroPEM) against the gravimetric method for PM_2.5_. Abbreviation: QT50 calibrated, QT50 calibrated by model, QT50 cf, QT50 calibrated by correction factor (CF), CF = 1 + 0.25 * RH * RH/(1 − RH), if RH>6.

**Fig 6:**
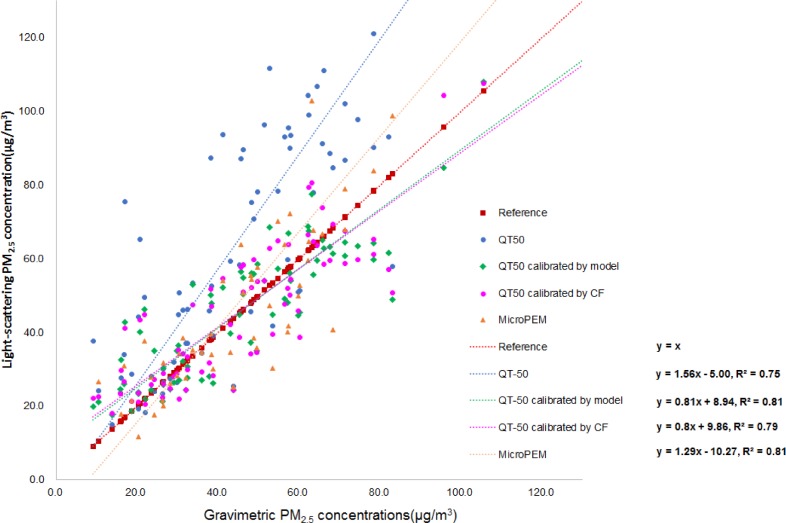
The original and the model-calibrated QT-50 PM_2.5_ as well as the MicroPEM measurements in comparison with the ideal Y(light scattering data) = X (gravimetric data) line. Y axis refers to PM_2.5_ concentration based on the light-scattering method, and X axis refers to PM_2.5_ concentration measured by gravimetric instrument. The red line Y = X is achieved when light-scattering measurements (QT50, MicroPEM) were in an ideally perfect agreement with the gravimetric measurement.

To test how the original PM_2.5_ data measured by QT-50 were improved after applying the calibration model, further comparison analyses were performed. Firstly, the median and the range of calibrated QT-50 data became closer to the level of the gravimetric data (Fig 5). By calculation, the ratio of calibrated QT-50 and gravimetric measurements decreased from 1.51(0.66) to 1.09(0.38). Secondly, the potential measurement bias by RH and temperature were significantly corrected. In the scattering plot of QT-50 after calibration, the PM_2.5_ concentration was not increased with the increase of RH anymore (Fig 3B), and the PM_2.5_ level was much closer to gravimetric measurement across different temperature (Fig 4B). Thirdly, by plotting the QT-50 data against the gravimetric data, the QT-50 calibration line was much closer to the theoretically ideal line of Y = X compared to the original QT-50 data line (Fig 6). The correlation between QT-50 and gravimetric measurement was improved by the increased adjusted R^2^ from 0.75 to 0.81 (Y = 0.81X+8.94). The MicroPEM data, which were considered as the reference light-scattering data, showed the adjusted R^2^ of 0.81 (Y = 1.29X-10.27).

## Discussion

In this study, a parallel 8-month sampling by both the light-scattering method (QT-50, MicroPEM) and the gravimetric method was performed. It was found that the light-scattering instrument overestimated the PM_2.5_ compared with the gravimetric method and the monitoring data were influenced by RH and temp. A calibration model (Y = 57.45 + 0.47 × X − 0.53 × RH − 0.41 × Temp) was further constructed for QT-50 by multiple linear regression against the gravimetric method with RH and temperature adjusted.

By comparing with the gravimetric method, the PM_2.5_ levels measured by QT-50 were on average 1.5 times higher. Further analyses suggested that RH was a major potential influencing factor and was adjusted in the final calibration model. In previous research [20–24], to control the influence of RH, a RH correction factor was used. The RH correction factor (CF) for light-scattering instrument was calculated as follows if the RH>60% [25]:

CorrectionFactor(CF)=1+0.25*RH*RH/(1−RH) (4)

In this study, we compared the data by using the RH correction factor when RH >60% with the calibrated data by regression models. By calibration using the CF and our regression model, the average QT-50 PM_2.5_ levels were 44.62 μg/m^3^ and 44.27 μg/m^3^ (Fig 5), respectively. The adjusted R^2^ was 0.79 and 0.81, respectively (Fig 6). The ratios between the CF corrected data/gravimetric data and the model calibrated data/gravimetric data were both 1.09. All these results indicated the high consistency between these two correction methods. Also, some other studies applied the model calibration. Ying Zhu et al showed the R^2^ between DustTrak (TSI Inc, Shoreview, MN, USA) and gravimetric measurement was 0.86[26], and Avril Challoner et al demonstrated the R^2^ of the above two different instruments was 0.50[27].

On the other hand, the data by MicroPEM instrument were calibrated for RH from a build-in program. So it was reasonable that no significant trend was observed between PM_2.5_ by MicroPEM and RH (Fig 3). The calibrated QT-50 data were comparable with the data by MicroPEM with the same adjusted R^2^ (Fig 6). Considering the convenience applied in the field tests and the higher cost-effectiveness, the QT-50 instrument can be superior with no need of filter weighing and treatment as necessitated by gravimetric methods.

Our calibration model was derived from a long-term field test covering a wide seasonal variation and meteorological conditions. In order to demonstrate the field calibration for a light-scattering sensor in a natural environment of interest, so that the findings and methodology may be extended and replicated by researchers who are interested in the utility of low-cost sensors such as QT-50. The method of field calibration for this class of sensors was also conducted in California at a regulatory monitoring site[28]. A good linear relationship between the PM_2.5_ mass concentration and the responses of low-cost optical PM sensors were also reported by previous studies [15, 28–30].

Calibrations should be conducted under different seasonal and environmental conditions to test how well this calibration model hold [31]. Limitations of our study should be noted. Firstly, the sample size in this study was relatively small. A larger number of sampling is needed to confirm our results. Secondly, the calibration study was performed in Shanghai, China, a place with relatively higher annual RH and temperature. More tests are needed to understand how stable of our calibration model application in other places with similar PM_2.5_ pollution patterns and climate characteristics.

## Conclusions

Light-scattering instruments such as QT-50 could overestimate the PM_2.5_ levels. The calibration model was set up after a long-term sampling covering a wide range of PM_2.5_ concentration, temperature and RH.

## Supporting information

S1 File The original dataset of PM_2.5_ mass concentration by 3 instruments, RH and temperature applied in the calibration models. (XLSX)
